# Comorbidities in Cushing’s disease

**DOI:** 10.1007/s11102-015-0645-6

**Published:** 2015-02-28

**Authors:** S. T. Sharma, L. K. Nieman, R. A. Feelders

**Affiliations:** 1 Program in Reproductive and Adult Endocrinology, Eunice Kennedy Shriver National Institute of Child Health and Human Development, National Institutes of Health, Bethesda, MD USA; 2 Division of Endocrinology, Department of Internal Medicine, Erasmus Medical Center, Gravendijkwal 230, 3015 CE Rotterdam, The Netherlands

**Keywords:** Cushing’s disease, Hypercortisolemia, Mortality, Morbidity

## Abstract

**Introduction:**

Cushing’s disease is a rare disorder characterized by overproduction of ACTH from a pituitary adenoma leading to hypercortisolemia that in turn leads to increased morbidity and mortality.

**Methods:**

Here we review the comorbidities associated with Cushing’s disease and their impact on quality of life and mortality.

**Results:**

Recent evidence suggests that correction of hypercortisolemia may not lead to complete resolution of comorbidities associated with this condition. In particular, increased cardiovascular risk may persist despite long-term remission of hypercortisolemia. This may be related to persistence of visceral adiposity, adverse adipokine profile, glucose intolerance, hypertension, dyslipidemia, atherosclerosis and a procoagulant phenotype. Prior prolonged exposure to glucocorticoids also may have irreversible effects on the central nervous system, leading to persistent cognitive and mood alterations. Osteoporosis and fractures, especially vertebral fractures, can further add to morbidity and a poor quality of life. Normalization of cortisol levels leads to significant improvement in comorbidities but long-term data regarding complete resolution are lacking and need further study.

**Conclusion:**

Early diagnosis and treatment of hypercortisolemia, aggressive management of comorbidities along with long-term follow-up is crucial for the optimal recovery of these patients.

## Introduction

Cushing’s disease (CD) is a rare disorder caused by overproduction of adrenocorticotrophin hormone (ACTH) by a pituitary adenoma that stimulates excess cortisol secretion from the adrenal glands [1]. Its incidence is 1.2–2.4 per million and prevalence is ~40 per million population [2, 3]. CD accounts for 75–80 % of cases with ACTH-dependent Cushing’s syndrome (CS).

The clinical presentation of CD can be highly variable and the diagnosis can often be challenging in cases with mild or cyclic hypercortisolism, especially given the overlap in symptoms in individuals with and without the disorder [1]. This may delay the diagnosis for 2–4 years [3–5]. Hypercortisolemia is associated with increased morbidity and mortality. Resection of the adenoma via transsphenoidal surgery (TSS) remains the optimal treatment. Medical therapy (steroidogenesis inhibitors, agents that decrease ACTH levels and glucocorticoid receptor antagonists) with or without pituitary radiotherapy may be needed [1] to normalize cortisol and/or its action. In addition, management of comorbidities (Table 1) is important because of increased cardiovascular risk despite remission [3–8]. We here review the comorbidities associated with CD and their impact on quality of life and mortality.

**Table 1:** Summary of comorbidities and their prevalence in active Cushing’s disease

Comorbidities	Prevalence	References
Cardiovascular/metabolic		
Obesity	32–41 %	[13–16]
Impaired glucose tolerance	21–64 %	[3, 7, 11, 15, 16]
Diabetes mellitus	20–47 %	[3, 7, 11, 15, 16]
Hypertension	55-85 %	[11, 15–18]
Dyslipidemia	38-71 %	[11, 16]
Hypercoagulopathy—hemostatic abnormalities	54 %	[22–25]
Hypercoagulopathy—venous thromboembolism	Incidence: 2.5–14.6/1000 persons/year	[25, 27]
Atherosclerotic changes	27–31 %	[11, 12, 15]
Cardiac structural and functional alterations	23–62 %	[19–21]
Bone		
Osteoporosis	38–50 %	[5, 34–36]
Fractures (vertebral and rib fractures)	15–50 %	[5, 34–36]
Kidney		
Nephrolithiasis	50 %	[41]
Psychiatric dysfunction		
Overall psychopathology	67 %	[42, 45]
Major depression	55–80 %	[43, 44]
Mood lability, irritability, anxiety, mania, psychosis, maladaptive personality		[42–46]
Cognitive impairment		[47–53]
Deficits in memory, verbal learning, spatial information, language and executive functioning		[52]
Subjective loss of brain volume	86 %	[50]
Hippocampal atrophy	27 %	[47]
White matter integrity changes		[53]
Poor health-related quality of life (HRQoL)		[54–58]

## Mortality in CD

Multiple studies show that the standardized mortality ratio (SMR) is increased in CD (1.7–4.8) [3–10], especially in patients with persistent hypercortisolism (3.7–4.2) compared to those in remission (1.8–3.17). SMR is higher in CD patients compared to those undergoing TSS for non-functioning pituitary macroadenomas (NFPA) (2.39 vs. 1.24) [6].

Cardiovascular and cerebrovascular events are the most common cause of death in CD [6, 9]. In one study, CD patients died of cardiovascular disease (n = 4), cerebrovascular disease (n = 1), malignancy (n = 1), and infectious diseases (n = 1). The average age at death (62.4 years) was significantly lower than that seen in the general Dutch population [6]. Thus, normalization of cortisol levels improves but does not normalize mortality compared to the general population.

## Comorbidities in CD (Table 1)

### Cardiovascular disease

Prolonged exposure to hypercortisolemia is associated with multiple cardiovascular risk factors that strongly impact morbidity and mortality. These risk factors include visceral adiposity, systemic arterial hypertension, impaired glucose tolerance, dyslipidemia and hypercoagulability [1].

Cardiovascular risk is increased in CD patients even 5 years after remission [11, 12]. Compared with controls, CD patients had significantly higher waist-to-hip ratio, diastolic blood pressure (BP), oral glucose tolerance test (OGTT)—stimulated glucose and insulin levels, total/HDL cholesterol ratio and fibrinogen levels. These patients had increased atherosclerotic carotid artery changes, with higher intima-media thickness and lower distensibility coefficient on ultrasound imaging. Atherosclerotic plaques were found in 26.7 % of CD patients and <4 % of controls [11].

Both children and adults in biochemical remission have abnormally high total body fat and visceral to subcutaneous fat ratios after a follow-up of 3–11 years [13, 14]. The adult patients also have increased inflammatory markers and decreased adiponectin levels [14]. This persistence of central adiposity and an unfavorable adipokine profile may link metabolic alterations and cardiovascular morbidity in CD.

Hypertension occurs in 55–85 % of CD patients [11, 15, 16], but resolves after remission in only 44–75 % [14, 16, 17]. Persistent hypertension despite remission may be related to microvessel remodeling and/or presence of underlying essential hypertension. Older age, longer duration of exposure to hypercortisolemia and a longer duration of untreated hypertension are associated with persistently elevated BP after biochemical cure [16–18]. This underlies the importance of early control of hypertension even while establishing the diagnosis of CD.

Cardiac structural and functional alterations, including left ventricular hypertrophy, concentric remodeling, and reduced mid-wall systolic performance with diastolic dysfunction, occurs in active CD [19–21]. They are more severe in hypertensive patients, suggesting an interaction between the deleterious effects of hypertension and cortisol excess per se [19]. With remission of hypercortisolemia, cardiac alterations significantly improve [19, 21] but may not normalize [19].

Patients with CD have an increased predisposition to thromboembolic events, especially in the perioperative period, likely related to a procoagulative phenotype due to cortisol excess [22–24]. In one systematic review, the incidence of venous thromboembolism (VTE) in CS was 2.5–3.1 per 1000 persons/year [25] compared to 1.0–2.0 per 1000 persons/year in the general population [26]. In a multicenter retrospective cohort study of 473 CS patients (360 CD), the incidence of VTE before treatment of hypercortisolemia was 14.6 per 1000 persons/year [27]. Additionally, the incidence of postoperative VTE was significantly greater compared to patients who underwent TSS for NFPAs (3.4 vs. 0 %) with the highest risk being in the first 2 months after surgery [27]. However, biochemical remission of hypercortisolemia with medical therapy (80 days) did not normalize hemostatic and fibrinolytic parameters [23]. At present, the duration and severity of risk for VTE following remission of hypercortisolism remain uncertain. There are no clear guidelines on the dose and duration of thromboprophylaxis either before or after surgery. Future long-term studies are needed to evaluate these issues.

### Insulin resistance, impaired glucose tolerance and diabetes mellitus

Insulin resistance is a well-known complication of CD. Glucocorticoid excess induces the expression of several key gluconeogenesis enzymes resulting in increased glucose production. It also impairs insulin sensitivity via direct interference with the insulin receptor signaling pathway as well as indirectly, through the stimulation of lipolysis and proteolysis [28]. The prevalence of overt diabetes mellitus is 20–47 % and that of impaired glucose tolerance is 21–64 % in CD [1, 11, 15, 16]. This may be an underestimation as not all patients undergo an OGTT, which is needed to diagnose impaired glucose tolerance when the fasting glucose is normal. The homeostatic model assessment (HOMA) shows decreased insulin sensitivity index (ISI) and increased insulin resistance in CD. Normal weight, overweight and obese patients have similar values, suggesting that these alterations result from hypercortisolemia and not obesity [14, 16]. Age, genetic predisposition and lifestyle also contribute significantly to the development of glucose intolerance in CD [29].

### Dyslipidemia

Lipid abnormalities (increased total and LDL cholesterol, triglycerides and total/HDL cholesterol ratio) occur in 38–71 % of CD patients [11, 16]. The multifactorial pathogenic mechanisms include direct and indirect actions of cortisol on lipolysis, free fatty acid production and turnover, very-low density lipoprotein synthesis and fatty accumulation in the liver. Insulin resistance, growth hormone deficiency and hypogonadism are also contributing factors [30]. Given the increased cardiovascular morbidity and mortality in CD, aggressive treatment of dyslipidemia is recommended [31]. Surgical remission is often associated with normalization of lipid abnormalities.

Management of dyslipidemia can be challenging in patients on medical therapy. Mitotane significantly increases cholesterol levels [32]. Drug interactions are common with ketoconazole, a potent inhibitor of cytochrome P450 3A4 (CYP3A4). Concomitant use of ketoconazole and certain statins (e.g. simvastatin and atorvastatin) that undergo metabolism via CYP3A4 can lead to significantly higher plasma levels of the statin, thus increasing the risk of complications and side-effects. In this setting, preference should be given to agents that are not metabolized by the CYP3A4 pathway (e.g. pravastatin) with close monitoring of liver function tests [31, 33].

### Osteoporosis, fractures and nephrolithiasis

The prevalence of fractures and osteoporosis as assessed by dual energy X-ray absorptiometry (DXA) has been reported to be 15–50 and 38–50 % respectively [5, 34–36], and may be a presenting feature of CD. These rates are likely an underestimation as not all CD patients undergo DXA scans, and asymptomatic vertebral and rib fractures can remain undiagnosed. The prevalence of vertebral and rib fractures is significantly higher in men compared to women [5]. This results from direct and indirect effects of glucocorticoids on bone, including decreased osteoblastic and increased osteoclastic activity, hypogonadism, growth hormone deficiency, reduced intestinal calcium absorption and increased urinary calcium excretion [37].

Normalization of cortisol levels has been associated with reversal of glucocorticoid-induced osteoporosis after 6–9 years [38, 39]. However, other shorter studies with 2–3 years follow-up show incomplete recovery of bone mineral density and quality of bone after remission [36, 40].

Nephrolithiasis is reported in 50 % of active CD patients compared to 6.5 % in age- and gender-matched controls. This is accompanied by hypercalciuria, hypocitraturia, hyperuricosuria and hyperoxalaturia [41], with amelioration of this lithogenic excretion pattern after remission. During remission, the prevalence of nephrolithiasis decreases (27.3 %) but remains higher compared to controls [41].

### Psychiatric dysfunction

Most (54–85 %) patients with CD have some psychiatric disturbance. Depression and irritability are most common emotional lability, mania, paranoia, acute psychosis, anxiety and panic attacks also occur [42, 43]. Many of these symptoms persist in the first few years after successful surgery [44–46]. However, overall psychopathology decreased significantly from 66.7 % during active CD to 53.6 % at 3 months, 36 % at 6 months and 24.1 % at 12 months after remission [44]. In another study, CD patients achieving long-term (mean 11 years) remission showed an increased prevalence of psychopathology and maladaptive personality traits compared to those with NFPAs and matched controls. These observations suggest irreversible effects of cortisol excess on the central nervous system rather than an effect of pituitary tumors or their treatment [46].

### Cognitive impairment

Chronic hypercortisolism is associated with cognitive dysfunction including impairment of memory, visual and spatial information, reasoning, verbal learning and language performance [47–53]. The hippocampus, amygdala and the cerebral cortex, structures important for cognitive and emotional functioning, are rich in glucocorticoid receptors and presumably particularly vulnerable to hypercortisolemia. Hippocampal atrophy was seen in 27 % of 12 active CS patients and correlated with performance on cognitive tests [47]. Hippocampal formation volume increased by up to 10 % in 22 CD patients ~16 months after surgical remission of hypercortisolemia [51]. In a case control study, apparent loss of brain volume was noted in 86 % of CD patients (14 % mild, 43 % moderate, 29 % severe), 100 % of adrenal CS patients and 10 % of controls. Normalization of cortisol levels was associated with partial reversal of cerebral atrophy [50]. van der Werff et al. reported widespread reductions in fractional anisotropy (marker of tissue microstructural organization) and changes of white matter integrity in 22 CD patients in long-term remission (mean 11.9 years) compared with matched controls, with changes in the uncinate fasciculus being related to the severity of depressive symptoms [53]. Recently Tiemensma et al. [52] reported significantly lower scores on cognitive assessment tests reflecting impaired memory and executive functioning, in 74 CD patients despite long-term remission (mean 13 years) compared with age-, gender- and education-matched controls. These data suggest that the structural changes in the brain induced by hypercortisolemia are at least partially reversible with normalization of cortisol levels. However, there may be a delay of several years from eucortisolemia to the resolution of cognitive deficits, and occasionally these may be irreversible.

## Health related quality of life (HRQoL)

HRQoL is significantly impaired in patients with CD [54–57]. In one study, the issues most often reported by CS patients with regards to the impact of the disease on their everyday life were fatigue and weakness (85 %), interference with family life and relationship with their partners (80 %), changes in physical appearance (63 %), emotional instability (61 %), impaired school/work performance (56 %), cognitive problems (49 %), depression (32 %) and sleeping difficulties (12 %) [51]. A prospective study of HRQol using the short-form 36 (SF-36) survey in 23 CD patients before and after TSS demonstrated that active CD was associated with low physical and mental summary scores with partial resolution after surgical remission of hypercortisolemia [55]. In a recent cross-sectional study of 102 patients with surgically treated CD (mean time since surgery 7.4 years), 92 % met the criteria for biochemical remission but only 80 % felt that they had been cured. These findings reflect the discordance between biochemical and self-assessed disease status and its impact on HRQoL in CD patients [57]. In a recent systematic review of quality of life impairments in patients with pituitary adenomas, biochemical remission of CD was associated with the smallest improvement in QoL measurements compared to those with other pituitary adenomas. Somatic factors (including hypopituitarism), psychological factors (illness perceptions), and health care environment (rural vs. urban) were identified as influencing factors [58].

## Economic burden of CD

Swearingen et al. analyzed data from a US administrative claims database for the period 2004–2008 with data on hospital admissions, physician visits, emergency room visits and medication use. The total health care costs for CD patients were fourfold greater than age- and gender-matched population controls and twofold higher than age- and gender-matched patients with NFPAs. Annual outpatient costs decreased significantly after surgical remission. In contrast, there was a significant increase in postoperative health care costs in those patients not in remission [59]. A more recent analysis of health care costs in CD based on 2010 US claims data estimated the total cost of care at $35,000/year [60]. These data along with the decreased working ability of CD patients and increase in sick leave highlight the significant economic burden placed on the patients as well as on the health care resources in CD.

## Conclusion

Although rare, CD is associated with a significant clinical, social, economic and quality of life burden. Uncontrolled hypercortisolemia is associated with metabolic, cardiovascular, cognitive and psychological alterations leading to increased mortality. Normalization of cortisol levels leads to significant improvement in these parameters and decreased mortality. However a growing body of evidence indicates that many risks and morbidities, in particular cardiovascular risk, persist for several years after remission. Larger studies with long-term follow-up are needed to elucidate this further. Early diagnosis and control of hypercortisolemia, aggressive management of comorbidities in a multidisciplinary setting and long-term follow-up are essential for optimal recovery in CD.
